# Pre-procedural image-guided versus non-image-guided ventricular tachycardia ablation—a review

**DOI:** 10.1007/s12471-020-01485-z

**Published:** 2020-09-15

**Authors:** A. A. Hendriks, Z. Kis, M. Glisic, W. M. Bramer, T. Szili-Torok

**Affiliations:** 1 Department of Electrophysiology, Erasmus MC, University Medical Center, Rotterdam, The Netherlands; 2 Department of Epidemiology, Erasmus MC, University Medical Center, Rotterdam, The Netherlands; 3 Medical Library, Erasmus MC, University Medical Center, Rotterdam, The Netherlands

**Keywords:** Magnetic resonance imaging, Computed tomography, Ventricular tachycardia, Catheter ablation, Meta-analysis

## Abstract

**Background:**

Magnetic resonance imaging and computed tomography in patients with ventricular tachycardia (VT) after myocardial infarction (MI) helps to delineate scar from healthy tissue. Image-guided VT ablation has not yet been studied on a large scale.

**Objective:**

The aim of the meta-analysis was to compare the long-term outcome of image-guided VT ablation with a conventional approach for VT after MI.

**Methods:**

Eight electronic bibliographic databases were searched to identify all relevant studies from 2012 until 2018. The search for scientific literature was performed for studies that described the outcome of VT ablation in patients with an ischaemic substrate. The outcome of image-guided ablation was compared with the outcome of conventional ablations.

**Results:**

Of the 2990 citations reviewed for eligibility, 38 articles—enrolling a total of 7748 patients—were included into the meta-analysis. Five articles included patients with image-guided ablation. VT-free survival was 82% [74–90] in the image-guided VT ablation versus 59% [54–64] in the conventional ablation group (*p* < 0.001) during a mean follow-up of 35 months. Overall survival was 94% [90–98] in the image-guided versus 82% [76–88] in the conventional VT ablation group (*p* < 0.001).

**Conclusions:**

Image-guided VT ablation in ischaemic VT was associated with a significant benefit in VT-free and overall survival as compared with conventional VT ablation. Visualising myocardial scar facilitates substrate-guided ablation procedures, pre-procedurally and by integrating imaging during the procedure, and may consequently improve long-term outcome.

**Electronic supplementary material:**

The online version of this article (10.1007/s12471-020-01485-z) contains supplementary material, which is available to authorized users.

## What’s new

- The use of imaging guidance in ventricular tachycardia (VT) ablation for patients with ischaemic heart disease is associated with higher VT-free survival.
- This is the first study that demonstrates a true large-scale benefit of visualising myocardial scar and integrating imaging in a VT ablation procedure.

## Introduction

Magnetic resonance imaging (MRI) and computed tomography advancing ventricular tachycardia (VT) ablation have an important role in diagnosing structural heart disease. In patients with VT after myocardial infarction (MI) these modalities of imaging help to identify, delineate and characterise the scar. Electroanatomical mapping can be used to define the scar and border zone. However, scar is 3dimensional and a voltage map is limited in spatial resolution [1]. Moreover, arrhythmogenic substrate may be found in heterogeneous tissue with normal voltages [2]. Channels that correlate with critical VT isthmuses can be identified by searching the scar for abnormal potentials. This may be time consuming and often incomplete. High-resolution MRI has been demonstrated to be able to delineate areas of surviving myocardial tissue within the scar that correlate with VT channels [3]. MRI preceding VT ablation can accurately predict recurrences in the presence of scar [4] and is a promising tool to identify ablation strategy in case of transmural scar [5].

Currently randomised and large scale trials lack on the long term outcome of image-guided VT ablation. The aim of the current meta-analysis is to perform a large scale analysis, comparing the long term outcome of image-guided VT ablation to a conventional VT ablation approach.

## Methods

### Data sources and search strategy

This review was conducted in accordance with the PRISMA and MOOSE guidelines (Appendix 1 and 2). The purpose of our study was to identify all studies that use imaging modalities to focus on scar that is performed prior to ablation for ischaemic VT. We searched Embase.com, Medline via Ovid, Web-of-science Core collection, the Cochrane Central registry of trials, Scopus, CINAHL via EBSCOhost and Google Scholar from January 2012 until January 2018. The search strategy was created with the assistance of a medical librarian (WB). The search strategy combined terms for ventricular tachycardia and catheter ablation, and terms for myocardial scar due to previous ischaemic injury. The search results were limited to English language articles. The detailed search methodology for all databases is provided in Appendix 3.

### Study selection and data extraction criteria

Studies were included if they: (i) were observational studies or randomised controlled trials (ii) reported on long-term follow-up of patients who underwent percutaneous catheter ablation for ischaemic VT, (iii) provided data on recurrences with a follow-up duration of >1 year. Articles that focused on patients with structural heart disease other than ischaemic scar were excluded. Also, if the studied population was heterogeneous and we were not able to extract the outcome of the patients with ischaemic VT, the study was excluded. Individual case reports, editorials, review articles and conference meeting abstracts were not included. We compared image-guided VT ablation with non-image-guided VT ablation. If imaging was performed preceding VT ablation but did not influence the ablation procedure, it was seen as non-image-guided VT ablation.

Two reviewers (AAH, ZK) independently evaluated the titles and abstracts according to the inclusion and exclusion criteria. For each potentially eligible study, two reviewers assessed the full-text. In cases of disagreement, a decision was made by consensus or, if necessary, a third reviewer (TSZT). was consulted. A predesigned data extraction form was used to collect relevant information on baseline characteristics, ablation method, imaging, procedural data and follow-up.

### Risk of bias assessments for the included clinical studies

The risk of bias within each individual study was evaluated by two reviewers (AAH, ZK) based on the nine-star Newcastle–Ottawa Scale (NOS) using three pre-defined domains namely: selection of participants, comparability and ascertainment of outcomes of interest. The NOS attributes a maximum of four points for selection, two points for comparability, and three points for outcome. Studies that received a score of nine points were judged to be at low risk of bias; studies that scored seven or eight stars were considered at medium risk; those that scored six or less were considered at high risk of bias (Appendix 4).

### Data synthesis and analysis

The unpaired Student’s t‑tests was used for demographic comparison of continuous variables between groups. We used metaprop command to pool proportions and we presented a weighted sub-group and overall pooled estimates with inverse-variance weights obtained from a random-effects model. Heterogeneity was quantified using the I^2^ statistic, classified as low (I^2^ ≤25%), moderate (I^2^ >25% and <75%), or high (I^2^ ≥75%). Additionally, Q‑statistic was used to assess the presence of heterogeneity. PQ statistic ≥0.05 was considered to indicate no significant heterogeneity among the included studies. Study characteristics including the location of the study, duration of the study, age, male sex, left ventricular function, the presence of VT storm at baseline and the percentage of patients using amiodarone were pre-specified as characteristics for assessment of heterogeneity and were evaluated using stratified analyses and random-effects meta-regression if 10 or more studies were included in the meta-analysis. Publication bias was evaluated through a funnel plot and asymmetry was assessed using the Egger’s test. All tests were two-tailed and *p*-values of 0.05 or less were considered statistically significant. STATA release 14 (Stata Corp, College Station, Texas) was used for all statistical analyses.

## Results

### Identification of relevant studies

**Fig. 1:**
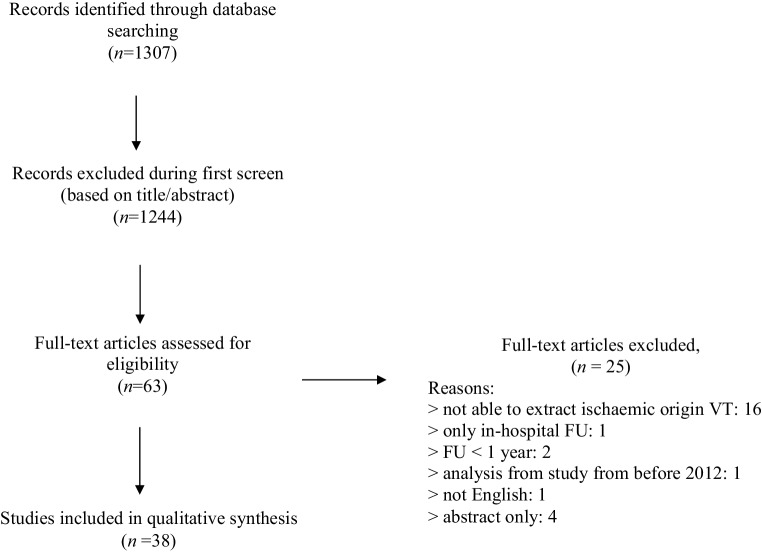
Flow chart of studies for outcome of ventricular tachycardia ablation. (*VT* ventricular tachycardia, *FU* follow-up)

The search strategy identified 2454 (1307 citations after excluding articles from before 2012), out of which 63 articles were found relevant following initial screening based on titles and abstracts. After full-text reading, 25 articles were further excluded based on extraction of ischaemic VT data and the follow-up criteria. A total of 38 articles were included that describe the outcome of VT ablation [2, 4–40]. Of the 38 articles five were image-guided [4–8]. Fig. 1 shows the selection process.

### General characteristics of the included studies

Tab. 1 shows the key characteristics of the included studies.

**Table 1:** Characteristics of included studies

Publication			Method of study	Patients with ischaemic VT	FU duration (months)
Author	Year	Country			
*A. Image-guided*					
Neijm [4]	2015	USA	OS, Co, SC	8	17
Acosta [5]	2016	Spain	OS, Co, SC	58	23
Yamashita [6]	2016	France	OS, Co, SC	67	17
Yamashita [7]	2016	France	OS, Co, SC	54	28
Andreu [8]	2017	Spain	Os, Co, SC	37	20
*B. Non-image-guided*					
Di Biase [9]	2012	Hungary, Italy, USA	OS, Co, MC	92	25
Dinov [10]	2012	Germany	OS, Co, SC	102	14
Arenal [11]	2013	Spain	OS, Co, SC	59	38
Ghanem [12]	2013	Egypt	OS, Co, SC	22	12
Tung [13]	2013	USA	OS, Co, SC	69	12
Aryana [15]	2014	USA	OS, Co, MC	36	19
Goya [16]	2014	Japan	OS, Co, SC	51	41
Mork [17]	2014	Denmark	OS, Co, SC	90	39
Saggu [18]	2014	India	OS, Co, SC	5	46
Silberbauer [19]	2014	Italy	OS, Co, SC	160	47
Tilz [20]	2014	Germany	OS, Co, SC	12	40
Avila [2]	2015	Spain	OS, Co, SC	46	32
Clemens [21]	2015	Czech Republic	OS, Co, SC	31	46
De Riva [22]	2015	The Netherlands	OS, Co, SC	91	23
Di Biase [23]	2015	China, Europa, USA	RCT, MC	118	12
Izguierdo [24]	2015	Spain	OS, Co, SC	50	13
Luther [25]	2015	UK	OS, Co, SC	24	24
Pioretti [26]	2015	USA	OS, Co, SC	87	54
Siontis [27]	2015	Europa, USA	OS, Co, MC	1412	56
Tsiarchis [26]	2015	Italy	OS, Co, SC	100	52
Tung [29]	2015	USA	OS, Co, MC	1095	12
Yokokowa [14]	2015	USA	OS, Co, SC	906	35
Acosta [30]	2016	Spain	OS, Co, SC	44	46
Dinov [31]	2016	Germany	OS, Co, SC	50	12
Frankel [32]	2016	Italy, Japan, USA	OS, Co, MC	1095	12
Fukunaga [33]	2016	Japan	OS, Co, SC	51	40
Ozcan [34]	2016	North America	OS, Co, SC	44	28
Sapp [35]	2016	Europe, USA	RCT, MC	132	28
Skoda [36]	2016	Czech Republic, Germany, USA	OS, Co, MC	53	12
Jin [37]	2017	China, Denmark	OS, Co, MC	54	17
Kuck [38]	2017	Germany	RCT, MC	60	28
Kuroki [39]	2017	Japan	OS, Co, MC	109	24
Tzou [40]	2017	USA, Japan	OS, co, MC	1174	12

*CO* cohort, *FU* follow-up, *MC* multi-centre, *ND* no data, *OS* observational study, *SC* single-centre, *RCT* randomised controlled trial, *VT* ventricular tachycardia

### Baseline characteristics

A total number of 7748 patients with VT from ischaemic scar were included in this meta-analysis (Tab. 2). Image-guided VT ablation had taken place in 224 patients. The majority of the non-image-guided articles were by authors from the USA, whereas the majority of the image-guided articles were by authors from Europe. The average age was 65 years and 89% was male. The average ejection fraction was 33%. Electrical storm was the reason for VT ablation in 16–100% of the population in the 23 studies that reported on it.

**Table 2:** Baseline characteristics of patients in included articles

		Number of studies	Image-guided	Non-image-guided	*p*-value
*Age*		38	64	66	0.07
*EF*		38	34	32	0.09
			(%)	(%)	
*Male*		36	94	90	0.40
*Infarct location*	Anterior	21	43	40	0.80
	Inferoposterior	20	52	46	0.66
*NYHA class III* *+* *IV*		18	11	34	0.07
*Electric storm*		23	49	36	0.45
*Diabetes mellitus*		22	19	30	0.13
*Hypertension*		27	70	60	0.11
*Amiodarone therapy*		34	68	54	0.41
*Prior VT ablation*		23	32	15	0.14
*Epicardial access*		34	37	6	<0.01
*ICD carrier*		31	55	94	0.02
			(min)	(min)	
*Procedural duration*		28	269 [250–]	220 [194–265]	0.09
*Radiofrequency time*		13	34 [32–]	38 [24–67]	0.93
*Radiation exposure*		24	54 [34–]	28 [15–38]	0.18

*EF* ejection fraction, *NYHA* New York Heart association, *ICD* implantable cardioverter defibrillator, *VT* ventricular tachycardia

Substrate ablation was applied in 50% of the included articles. Eighty percent of the image-guided VT ablation articles primarily used a substrate approach. In 7 articles, targeted ablation was applied and in one article [9] there was a direct comparison between a targeted and a substrate approach. Three articles, all non-image-guided, described ablation using remote magnetic navigation (49–100% of the patients). Seven articles, all non-image-guided, commented on using assist devices in patients with non-haemodynamically tolerable VTs. Fifty-five percent of the patients in the image-guided ablation group and 94% in the conventional VT ablation group, were ICD carriers (*p* = 0.02) at baseline. Fifty-eight percent had reported amiodarone use at the time of ablation.

No studies were judged at low bias of risk. Among the observational studies, studies were judged to be at median or high risk of bias. Among the randomised controlled trials, studies were all judged at median bias of risk.

### Techniques used in image-guided VT ablation

Various types of image-guided ablation were reported. One article reported on the use of imaging for planning the ablation strategy [5], 2 articles on imaging integrated in the ablation procedure [7, 8], and 2 articles reported on doing both [4, 6]. One of the articles that integrated imaging in the procedure used Automatic Detection of Arrhythmic Substrate (ADAS) [8].

### Procedural difference in characteristics between image-guided and non-image-guided VT ablation

Characteristics between the image-guided and non-image-guided VT ablations were similar except for a significant difference in percentage of patients who had epicardial access; 37 in the image-guided versus 6 in the non-image-guided VT ablation (*p* < 0.01) (Tab. 2).

### Procedural data

Procedural duration was on average 4.5 h in the image-guided ablation versus 3.7 h in the conventional ablation procedure (*p* = 0.09). There was no significant difference between radiofrequency time and fluoroscopy time in image-guided VT ablation versus conventional ablation (Tab. 2).

### Long-term outcome in VT ablation

Sixty-one percent (interquartile range [IQR] 54–67) of the patients were free of VT recurrences during a mean follow-up duration of 35 months with an overall survival of 84% (IQR 80–88).

### Outcome of image-guided VT ablation

**Fig. 2:**
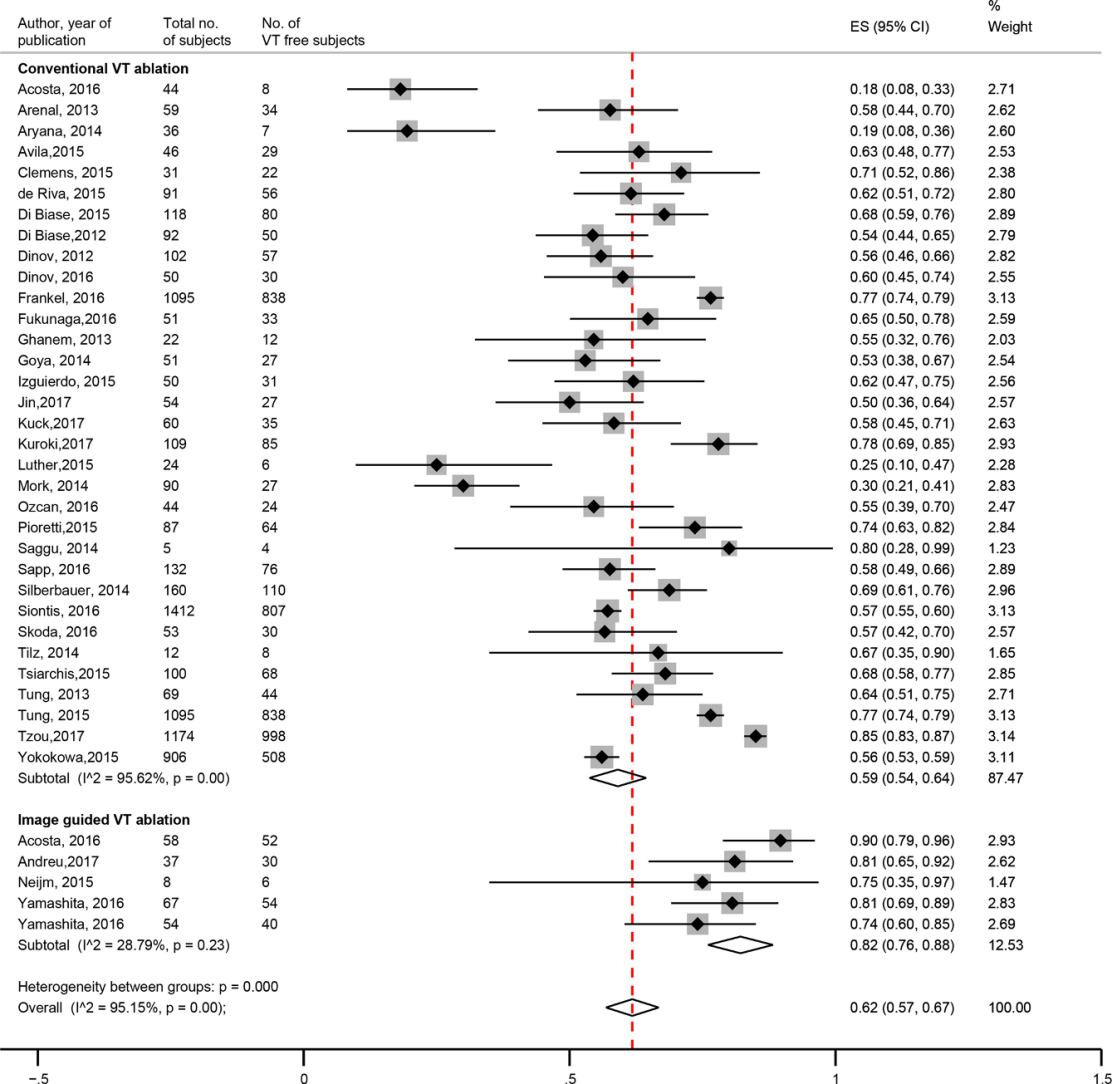
Forest plot of VT-free survival—image-guided versus non-image-guided. (*CI* confidence interval, *ES* effect size, *VT* ventricular tachycardia)

**Fig. 3:**
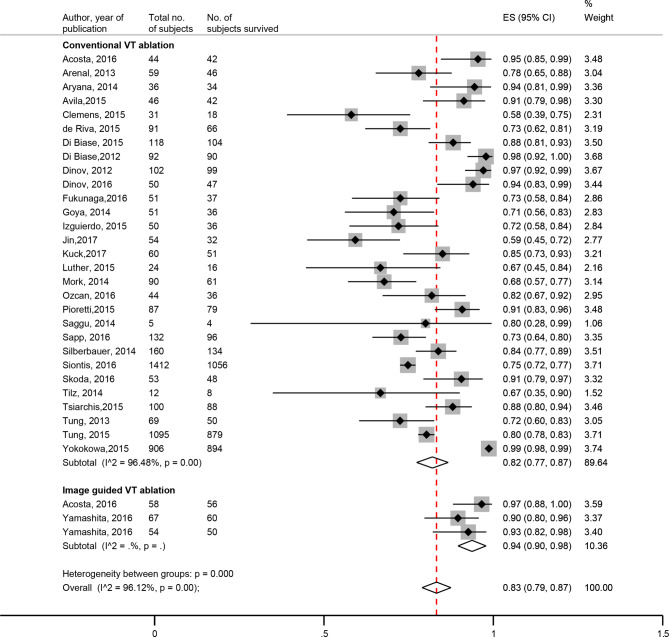
Forest plot survival—image-guided versus non-image-guided. (*CI* confidence interval, *ES* effect size, *VT* ventricular tachycardia)

The image-guided VT ablation reported a higher VT-free survival of 82% [IQR 76–88] compared with the non-image-guided VT ablations (59%, IQR 54–64, *p* < 0.001) (Fig. 2). Overall survival was 94% (IQR 90–98) in the image-guided versus 82% (IQR 77–87) in the conventional VT ablation (*p* < 0.001) (Fig. 3). High between-study heterogeneity (random effects model i^2^ 93.56%, *p* < 0.001) could not be explained by any of the investigated between-study characteristics (Supplementary file 1).

## Discussion

The current meta-analysis shows an improved VT-free and overall survival in patients with ischaemic VT by using image-guided VT ablation compared with conventional VT ablation. This is the first study that demonstrates true large-scale benefit. Visualising myocardial scar and integrating imaging in the procedure facilitates VT ablation by focussing on the area of interest and providing more accurate substrate characterisation.

### Imaging-derived scar versus electroanatomical mapping

Generally, there is a good correlation between bipolar voltage mapping and computed tomography and MRI-derived scar [6, 41]. Increased transmurality of the scar on MRI correlated well with reduced bipolar low voltage on the endocardium [42], suggesting the presence of low voltage in the epicardium.

However, voltage mapping may fail to accurately delineate the extent of diseased myocardium because of limitations such as catheter contact issues, reduced sensitivity to far-field signal of the mid-myocardium [43], and the interposition of epicardial fat [44]. Epicardial fat may lead to false-negative low voltage on epicardial voltage mapping, computed tomography accurately visualises epicardial fat and differentiates it from scar [44]. Scar is a 3-dimensional structure and consequently a voltage map has a limited spatial resolution. Moreover, a bipolar voltage map may show absence of low voltage in the presence of an intramural scar and, therefore, may be missed. It is potentially unmasked with unipolar recordings, but may be best visualised with MRI. In the presence of an intramural scar, VT recurrences occur more frequently [4]. Recognising the presence of intramural scar, high-output endocardial ablation, bipolar ablation or a radiofrequency needle ablation catheter may reach the mid-myocardium and successfully ablate the VT circuit [45].

### Limitations of MRI

The use of cardiac MRI in VT ablation has certain limitations. Currently there is no consensus on, nor standardisation of, image postprocessing [46]. Another limitation is the presence of artefacts that derive from ICDs, most commonly affecting the basal left anterior free wall. The presence of devices not only limits the interpretations of scar tissue on MRI, but also affects the reliability of contrast-enhanced imaging due to provoked hyper-intense off-resonance artefacts mimicking scar tissue [47]. The wideband inversion recovery late gadolinium enhancement (LGE) MRI technique can potentially overcome this type of artefact [48].

Furthermore, errors can arise with image integration as well. Geometry can change due to respiratory and cardiac motion, and conformational changes can occur between the time of MRI and the ablation procedure due to, for example, differences in volume or rhythm [47]. Partial-volume effects on the standard thickness short-axis slices, for example, can lead to overestimation of border zone areas [49]. In EP procedures this is a known phenomenon. During electroanatomical mapping (EAM), the mean maximum amplitude of cardiac and respiratory motion was 10.2 ± 2.7 mm and 8.8 ± 2.3 mm respectively [50]. This may be especially critical in the identification of conduction channels.

Using real-time MRI minimalises conformational changes. Non-contrast-enhanced T1-weighted imaging with long T1 decay times is promising as it was shown to be an effective method for visualising necrosis within radiofrequency ablation lesions. Enhancement is more specific and stationary than that from contrast LGE MRI. Scar tissue appears dark in the non-contrast-enhanced images, allowing to differentiate between acute radiofrequency ablations and chronic scar [51]. Cardiac motion correction by cardiac triggering improves precision in myocardial T1 mapping [52].

### Image-guided ablation strategy

Epicardial ablation in ischaemic VT is usually restricted to patients with previous failed endocardial ablation attempts. Yet, there is a relation between complete VT substrate elimination and better ablation outcomes [11]. The importance of complete substrate ablation is in the assumption that substrate not related to clinical or inducible VT can activate and become a VT isthmus during follow-up. Epicardial border zone channels in post-MI transmural scar are seen in 63% [3].

However, if epicardial ablation is used as a first-line ablation a significant proportion of patients undergoing epicardial mapping do not exhibit an epicardial arrhythmogenic substrate [9]. Which is why tools are needed to avoid unnecessary pericardial explorations. Acosta et al. showed that endocardial ablation in patients with a transmural scar on MRI resulted in a significantly lower recurrence-free survival compared with complete substrate ablation [5].

### Scar characterisation

A large area of scar heterogeneity predicts recurrence of VT after VT ablation [4]. Furthermore, successful ablation appears to be in localised areas of heterogeneity, and incomplete ablation in these areas predicts VT recurrence in animal models [53]. The delayed components of the conducting channel electrograms reflect the presence and activation of viable fibres embedded in fibrosis [54]. Border zone channels display a 3D structure within the myocardial wall that can be depicted by contrast-enhanced MRI [3]. Critical sites of ischaemic VT are confined to areas of high signal intensity. Channels on MRI correlate with areas of survival myocardial tissue which help to better locate the target ablation sites and find critical channels in the areas of normal voltage [8, 55]. Identification of conductive channels on EAM aided by pixel intensity maps improved when based on 3D imaging with 1.4 × 1.4 × 1.4-mm resolution compared with conventional 2‑dimensional clinical imaging with 5‑mm slice thickness [3]. Identification of conduction channels in the electroanatomical map can be improved when using a cut-off value of 60% of the maximum pixel signal intensity, both for core and border zone. Using computed tomography, thicker ridges within areas of pronounced wall thinning in the scar, seen as relatively preserved wall thickness, are recognised as the arrhythmogenic substrate of scar-related VT [56].

Critical VT isthmus sites in patient with prior MI are located in close proximity to the area on MRI where transition between >75% transmural scar and the core border zone occurs [57]. Critical isthmus sites around the core border zone transition suggest that the signal intensity threshold of a maximal 50% may indicate a critical mix between fibrosis and viable myocytes that allow for slow conduction and thereby, re-entrant VT. Currently, however, 3D imaging and postprocessing methods may still be limited at detecting fractionated and late potential regions within EAM dense scar [3].

### Image integration

Real-time integration of VT substrate helps focusing on diseased versus healthy areas of the myocardium. Yamashita et al. showed that despite a similar number of mapping points a higher number of local abnormal ventricular activities (LAVA) sites could be identified in patients who had ablation guided by imaging data [6]. More efficient mapping focuses towards the critical areas when guided by imaging data and leads to better long-term freedom of VT [7, 8].

### Clinical implications

Imaging guidance for VT ablation is not mentioned in the current ventricular arrhythmia guidelines [58]. The current meta-analysis suggests benefit in VT-free and overall survival by the use of image guidance in VT ablation for patients with ischaemic heart disease. Larger-scale randomised studies are needed to confirm our results. Furthermore, we are in need of studies that teach us about the cost-effectiveness of image-guided VT ablation.

### Limitations

Despite the fact that this is the largest image-guided VT ablation cohort so far, there are several limitations to note, some inherent to performing a meta-analysis. First, *s*ome data on patient level was unavailable in the included studies, which precluded a detailed evaluation to identify the impact of particular baseline demographic characteristics (i.e., number of ICD shocks before the ablation), type of imaging used (computed tomography or contrast-enhanced cardiac magnetic resonance) and procedural factors (use of magnetic navigation or contact force) on the outcome of freedom of VT. Additionally, we were not able to extract data on the correlation between ablation strategy (substrate versus targeted arrhythmia approach) and long-term outcomes in all of the eligible studies. There were significantly less patients with an ICD at the time of inclusion in the image-guided group even though ejection fraction was similar. A possible explanation for the low percentage of patients who had an ICD implanted at baseline is selection bias, patients without an implanted ICD during their presentation with VT were possibly more prone to undergo MRI before ablation. ICD patients benefit from a continuous monitoring system, it could influence the detection of VT during follow-up. We cannot exclude that an ICD was implanted during follow-up. Higher VT-free and overall survival has been seen in patients treated with a substrate approach including epicardial ablation compared with a limited ablation [9]. Patients with image-guided VT ablation more often had a substrate approach and had a higher percentage of epicardial access, which, in itself, could be an explanation for the lower number of recurrences in this group. Yet, determining an ablation strategy is one of the potential benefits from image-guided VT ablations [5]. There was minimal publication bias as indicated by conventional funnel plots and Egger test (Supplementary file 2), however, these approaches are limited by their qualitative nature. The majority of the included studies were observational in nature, with higher risk of selection bias. Randomised controlled trials are needed to convince the effectiveness of imaging data for VT ablation procedures. Limited scanning capacity and additional costs could hinder implementation. Currently, we cannot be confident that our results are merely a reflection of the contribution of image-guided ablation.

## Conclusion

Image-guided VT ablation was found to be associated with a significant benefit in VT-free and overall survival as compared to conventional VT ablation. Visualising myocardial scar may facilitate substrate-guided ablation procedures, pre-procedurally and by integrating imaging in the procedure, and consequently may improve long-term outcome.

## Caption Electronic Supplementary Material

Appendix 1: PRISMA checklist. Appendix 2: MOOSE checklist. Appendix 3: data search. Appendix 4: Newcastle- Ottawa Quality Assessment Scale

Supplementary files 1 and 2
