# Supplementary material for: Pre-procedural image-guided versus non-image-guided ventricular tachycardia ablation—a review
Source: Neth Heart J. 2020 Sep 15;28(11):573–83. doi: 10.1007/s12471-020-01485-z (PMC7596120; doi:10.1007/s12471-020-01485-z)
Supplement: Supplementary file 1 — Appendix 1: PRISMA checklist. Appendix 2: MOOSE checklist. Appendix 3: data search. Appendix 4: Newcastle- Ottawa Quality Assessment Scale [file 12471_2020_1485_MOESM1_ESM.docx]

**Appendix 1.** PRISMA checklist

| **Checklist item** |
| --- |

| **Section/topic** | **#** |  | **Reported on page #** |
| --- | --- | --- | --- |
| **TITLE** | | |  |
| Title | 1 | Identify the report as a systematic review and a meta-analysis | 1 |
| **ABSTRACT** | | |  |
| Structured summary | 2 | Provide a structured summary including, as applicable: background; objectives; data sources; study eligibility criteria, participants, and interventions; study appraisal and synthesis methods; results; limitations; conclusions and implications of key findings; systematic review registration number. | 2 |
| **INTRODUCTION** | | |  |
| Rationale | 3 | Describe the rationale for the review in the context of what is already known. | 6 |
| Objectives | 4 | Provide an explicit statement of questions being addressed with reference to participants, interventions, comparisons, outcomes, and study design (PICOS). | 6 |
| **METHODS** | | |  |
| Protocol and registration | 5 | Indicate if a review protocol exists, if and where it can be accessed (e.g., Web address), and, if available, provide registration information including registration number. | NA |
| Eligibility criteria | 6 | Specify study characteristics (e.g., PICOS, length of follow-up) and report characteristics used as criteria for eligibility, giving rationale. | 7 |
| Information sources | 7 | Describe all information sources (e.g., databases with dates of coverage, contact with study authors to identify additional studies) in the search and date last searched. | 7 |
| Search | 8 | Present full electronic search strategy for at least one database, including any limits used, such that it could be repeated. | 7 and Appendix 3 |
| Study selection | 9 | State the process for selecting studies (i.e., screening, eligibility, included in systematic review) | 7 |
| Data collection process | 10 | Describe method of data extraction from reports (e.g., piloted forms, independently, in duplicate) and any processes for obtaining and confirming data from investigators. | 7 |
| Data items | 11 | List and define all variables for which data were sought (e.g., PICOS, funding sources) and any assumptions and simplifications made. | 7 |
| Risk of bias in individual studies | 12 | Describe methods used for assessing risk of bias of individual studies (including specification of whether this was done at the study or outcome level), and how this information is to be used in any data synthesis. | 8,Appendix 4, Suppl. 1 |
| Summary measures | 13 | State the principal summary measures | 9 |
| Synthesis of results | 14 | Describe the methods of handling data and combining results of studies, if done, including measures of consistency (e.g., I2) for each meta-analysis. | NA |
| Risk of bias across studies | 15 | Specify any assessment of risk of bias that may affect the cumulative evidence (e.g., publication bias, selective reporting within studies). | 5, Suppl. 1 |
| **RESULTS** | | |  |
| Study selection | 17 | Give numbers of studies screened, assessed for eligibility, and included in the review, with reasons for exclusions at each stage, ideally with a flow diagram. | 6-8  Figure 1 |
| Study characteristics | 18 | For each study, present characteristics for which data were extracted (e.g., study size, PICOS, follow-up period) and provide the citations. | 8-11  Table 1,2 |
| Risk of bias within studies | 19 | Present data on risk of bias of each study and, if available, any outcome level assessment (see item 12). | 9  Suppl.1 |
| Results of individual studies | 20 | For all outcomes considered (benefits or harms), present, for each study: (a) simple summary data for each intervention group (b) effect estimates and confidence intervals, ideally with a forest plot. | 8-11  Table 1-2 |
| Synthesis of results | 21 | Present results of each meta-analysis done, including confidence intervals and measures of consistency. | Fig. 2 |
| Risk of bias across studies | 22 | Present results of any assessment of risk of bias across studies (see Item 15). | Suppl1 |
| Additional analysis | 23 | Give results of additional analyses, if done (e.g., sensitivity or subgroup analyses, meta-regression [see Item 16]). | Suppl1 |
| **DISCUSSION** | | |  |
| Summary of evidence | 24 | Summarize the main findings including the strength of evidence for each main outcome; consider their relevance to key groups (e.g., healthcare providers, users, and policy makers). | 10-14 |
| Limitations | 25 | Discuss limitations at study and outcome level (e.g., risk of bias), and at review-level (e.g., incomplete retrieval of identified research, reporting bias). | 13, 14 |
| Conclusions | 26 | Provide a general interpretation of the results in the context of other evidence, and implications for future research. | 11-13 |
| **FUNDING** | | |  |
| Funding | 27 | Describe sources of funding for the systematic review and other support (e.g., supply of data); role of funders for the systematic review. | 1 |

**Appendix 2.:** MOOSE checklist

| **Criteria** | | **Brief description of how the criteria were handled in the meta-analysis** |
| --- | --- | --- |
| **Reporting of background should include** | |  |
| √ | Problem definition | Magnetic resonance imaging (MRI) and computed tomography in patients with ventricular tachycardia (VT) after myocardial infarction helps to delineate scar from healthy tissue. MRI preceding VT ablation can prognosticate an increased recurrence rate in the presence of intramural scar and is a promising tool to identify an epicardial ablation strategy in case of transmural scar. No meta-analysis that compares the long-term outcome of imaging guided ventricular tachycardia ablation to conventional ventricular tachycardia ablation, has been published. |
| √ | Hypothesis statement | The usage of imaging guided ventricular tachycardia ablation improves long-term freedom from ventricular tachycardia recurrence as compared to conventional ventricular tachycardia ablation. |
| √ | Description of study outcomes | We included studies that estimated the outcome of ventricular tachycardia ablation in patient with an ischaemic substrate |
| √ | Type of exposure or intervention used | VT ablation with and without the guidance of imaging |
| √ | Type of study designs used | Eligible study designs included randomized controlled trials (RCTs), cohort, case-control studies. |
| √ | Study population | Only studies carried out in adults (>18 years old) were included. |
| **Reporting of search strategy should include** | |  |
| √ | Qualifications of searchers | The credentials of the investigators are indicated in the authors list. |
| √ | Search strategy, including time period included in the synthesis and keywords | Search strategy and time periods are detailed in page 7 of the manuscript and in Figure 1 and the full search strategy is available in Appendix 3. |
| √ | Databases and registries searched | Medline, Embase, Google Scholar, Scopus, CINAHL EBSCOhost, Web-of-Science, Cochrane Central |
| √ | Search software used, name and version, including special features | We did not employ a search software. Endnote was used. to merge retrieved citations and eliminate duplications. |
| √ | Use of hand searching | We hand-searched bibliographies of retrieved systematic reviews and meta-analysis for additional references. |
| √ | List of citations located and those excluded, including justifications | Details of the literature search process are outlined in the flow chart. Citations for the included studies are included in the text and table 1. The citation list for excluded studies is available upon request. |
| √ | Method of addressing articles published in languages other than English | We placed restrictions to English language. |
| √ | Method of handling abstracts and unpublished studies | Systematic reviews were used to identify further references. |
| √ | Description of any contact with authors | Authors of included studies were contacted to retrieve missing full texts and to identify any missing studies. |
| **Reporting of methods should include** | |  |
| √ | Description of relevance or appropriateness of studies assembled for assessing the hypothesis to be tested | Detailed inclusion and exclusion criteria were described in the methods section. |
| √ | Rationale for the selection and coding of data | A predesigned data collection form was prepared to extract the relevant information from the included full texts, including study design, imaging technique, ablation strategy and whether endo/epicardial ablation was performed in a first set-up. |
| √ | Assessment of confounding | We performed qualitative analyses to evaluate differences between studies |
| √ | Assessment of study quality, including blinding of quality assessors; stratification or regression on possible predictors of study results | We used the Newcastle- Ottawa Scale (NOS) to evaluate the quality of cross-sectional, case-control and cohort studies included in this review. |
| √ | Assessment of heterogeneity | We were able to pool the data statistically. |
| √ | Description of statistical methods in sufficient detail to be replicated | Under Data Synthesis and Analysis there is a detailed description of the statistical analyses. |
| √ | Provision of appropriate tables and graphics | We included 2 main tables, 3graphs, 4 appendices, and 2 supplements |
| **Reporting of results should include** | |  |
| √ | Graph summarizing individual study estimates and overall estimate | Figure 2 and 3 |
| √ | Table giving descriptive information for each study included | Tables 1 and 2 |
| √ | Results of sensitivity testing | Supplement 1 |
| √ | Indication of statistical uncertainty of findings | 95% confidence intervals or SD’s were presented if available |
| **Reporting of discussion should include** | |  |
| √ | Quantitative assessment of bias | Not applicable |
| √ | Justification for exclusion | We excluded studies that had no or an unclear definition of outcome, or data extraction was not feasible. |
| √ | Assessment of quality of included studies | We used the Newcastle- Ottawa Scale (NOS) to evaluate the quality of cross-sectional, case-control and cohort studies included in this review. |
| **Reporting of conclusions should include** | |  |
| √ | Consideration of alternative explanations for observed results | Due to the lack of standardisation of follow-up, it is difficult to provide unifying statements within a larger number of manuscripts hampered by large levels of heterogeneity. |
| √ | Generalization of the conclusions | The generalizability of our findings has been enhanced by the involvement of data, including USA and Europe. However, there is a clear lack of evidence from the African and most of the West Pacific Region. |
| √ | Guidelines for future research | Further work is necessary to standardize the follow-up of patients who underwent image guided ventricular tachycardia ablation |
| √ | Disclosure of funding source | Nothing to disclose. |

**Appendix 3.:** data search

**Embase.com**

('heart ventricle arrhythmia'/exp OR (((ventric* OR idioventric* OR rvot OR lv OR rv OR lvot) NEAR/3 (tachycard* OR tachyarrhythm* OR fibrillat* OR arrhythm*)) OR vt):ab,ti) AND ('ablation therapy'/exp OR 'ablation catheter'/de OR 'catheter ablation'/de OR 'radiofrequency ablation'/de OR 'radiofrequency ablation device'/de OR 'cryoablation'/de OR (ablati* OR cryoablati* OR rfa):ab,ti) AND ('ischemia'/de OR 'ischemic heart disease'/exp OR infarction/exp OR 'heart muscle ischemia'/exp OR scar/de OR (ischemi* OR ischaemi* OR infarct* OR postinfarct* OR scar ):ab,ti) NOT ([animals]/lim NOT [humans]/lim) NOT ([Conference Abstract]/lim OR [Letter]/lim OR [Note]/lim OR [Editorial]/lim) AND [english]/lim

**Medline Ovid**

(("Arrhythmias, Cardiac "/ AND "Heart Ventricles"/) OR "Ventricular Fibrillation"/ OR "Tachycardia, Ventricular"/ OR (((ventric* OR idioventric* OR rvot OR lv OR rv OR lvot) ADJ3 (tachycard* OR tachyarrhythm* OR fibrillat* OR arrhythm*)) OR vt).ab,ti,kf.) AND ("Ablation Techniques"/ OR "Catheter Ablation"/ OR (ablati* OR cryoablati* OR rfa).ab,ti,kf.) AND ("ischemia"/ OR "Myocardial Ischemia"/ OR "Myocardial Infarction"/ OR Cicatrix/ OR (ischemi* OR ischaemi* OR infarct* OR postinfarct* OR scar ).ab,ti,kf.) NOT (exp animals/ NOT humans/) NOT (letter OR news OR comment OR editorial OR congresses OR abstracts).pt. AND english.la.

**Cochrane CENTRAL**

((((ventric* OR idioventric* OR rvot OR lv OR rv OR lvot) NEAR/3 (tachycard* OR tachyarrhythm* OR fibrillat* OR arrhythm*)) OR vt):ab,ti) AND ((ablati* OR cryoablati* OR rfa):ab,ti) AND ((ischemi* OR ischaemi* OR infarct* OR postinfarct* OR scar ):ab,ti)

**Web of Science**

TS=(((((ventric* OR idioventric* OR rvot OR lv OR rv OR lvot) NEAR/2 (tachycard* OR tachyarrhythm* OR fibrillat* OR arrhythm*)) OR vt)) AND ((ablati* OR cryoablati* OR rfa)) AND ((ischemi* OR ischaemi* OR infarct* OR postinfarct* OR scar ))) AND DT=(article) AND LA=(english)

**Google scholar**

"ventricle|ventricular|rvot|lv|rv|lvot tachycardia|tachyarrhythmia|arrhythmia" ablation|cryoablation|rfa ischemia|ischaemia|ischemic|ischaemic|infarction|infarct

Imaging VT studies

**Embase.com**

('heart ventricle arrhythmia'/exp OR (((ventric* OR idioventric* OR rvot OR lv OR rv OR lvot) NEAR/3 (tachycard* OR tachyarrhythm* OR fibrillat* OR arrhythm*)) OR vt):ab,ti) AND ('ablation therapy'/exp OR 'ablation catheter'/de OR 'catheter ablation'/de OR 'radiofrequency ablation'/de OR 'radiofrequency ablation device'/de OR 'cryoablation'/de OR (ablati* OR cryoablati* OR rfa):ab,ti) AND ('imaging'/de OR 'cardiac imaging'/de OR 'diagnostic imaging'/de OR 'nuclear magnetic resonance imaging'/exp OR 'nuclear magnetic resonance'/de OR 'computer assisted tomography'/exp OR 'positron emission tomography'/de OR (imaging OR image* OR (magnet* NEAR/3 resonan*) OR ceMRI OR ceMR OR mri OR nmr OR mr OR (comput* NEAR/3 tomograph*) OR ct OR ((cat OR pet) NEAR/3 scan*) OR cmr OR cmri OR ((positron* OR emissi*) NEAR/3 tomogra*)):ab,ti) NOT ([animals]/lim NOT [humans]/lim)

**Medline Ovid**

("Tachycardia, Ventricular"/ OR (((ventric* OR idioventric* OR rvot OR lv OR rv OR lvot) ADJ3 (tachycard* OR tachyarrhythm* OR fibrillat* OR arrhythm*)) OR vt).ab,ti.) AND ("Ablation Techniques"/ OR "Catheter Ablation"/ OR (ablati* OR cryoablati* OR rfa).ab,ti.) AND ("Cardiac Imaging Techniques"/ OR "Diagnostic Imaging"/ OR exp "Magnetic Resonance Imaging"/ OR exp "Tomography, X-Ray Computed"/ OR exp "Tomography, Emission-Computed"/ OR (imaging OR image* OR (magnet* ADJ3 resonan*) OR ceMRI OR ceMR OR mri OR nmr OR mr OR (comput* ADJ3 tomograph*) OR ct OR ((cat OR pet) ADJ3 scan*) OR cmr OR cmri OR ((positron* OR emissi*) ADJ3 tomogra*)).ab,ti.) NOT (exp animals/ NOT humans/)

**Cochrane**

((((ventric* OR idioventric* OR rvot OR lv OR rv OR lvot) NEAR/3 (tachycard* OR tachyarrhythm* OR fibrillat* OR arrhythm*)) OR vt):ab,ti) AND ((ablati* OR cryoablati* OR rfa):ab,ti) AND ((imaging OR image* OR (magnet* NEAR/3 resonan*) OR ceMRI OR ceMR OR mri OR nmr OR mr OR (comput* NEAR/3 tomograph*) OR ct OR ((cat OR pet) NEAR/3 scan*) OR cmr OR cmri OR ((positron* OR emissi*) NEAR/3 tomogra*)):ab,ti)

**Web of science**

TS=(((((ventric* OR idioventric* OR rvot OR lv OR rv OR lvot) NEAR/2 (tachycard* OR tachyarrhythm* OR fibrillat* OR arrhythm*)) OR vt)) AND ((ablati* OR cryoablati* OR rfa)) AND ((imaging OR image* OR (magnet* NEAR/2 resonan*) OR ceMRI OR ceMR OR mri OR nmr OR mr OR (comput* NEAR/2 tomograph*) OR ct OR ((cat OR pet) NEAR/2 scan*) OR cmr OR cmri OR ((positron* OR emissi*) NEAR/2 tomogra*))) )

**Scopus**

TITLE-ABS-KEY(((((ventric* OR idioventric* OR rvot OR lv OR rv OR lvot) W/2 (tachycard* OR tachyarrhythm* OR fibrillat* OR arrhythm*)) OR vt)) AND ((ablati* OR cryoablati* OR rfa)) AND ((imaging OR image* OR (magnet* W/2 resonan*) OR ceMRI OR ceMR OR mri OR nmr OR mr OR (comput* W/2 tomograph*) OR ct OR ((cat OR pet) W/2 scan*) OR cmr OR cmri OR ((positron* OR emissi*) W/2 tomogra*))) )

**CINAHL EBSCOhost**

(MH "Tachycardia, Ventricular" OR TI (((ventric* OR idioventric* OR rvot OR lv OR rv OR lvot) N2 (tachycard* OR tachyarrhythm* OR fibrillat* OR arrhythm*)) OR vt) OR AB (((ventric* OR idioventric* OR rvot OR lv OR rv OR lvot) N2 (tachycard* OR tachyarrhythm* OR fibrillat* OR arrhythm*)) OR vt)) AND (MH "Ablation Techniques" OR MH "Catheter Ablation" OR TI (ablati* OR cryoablati* OR rfa) OR AB (ablati* OR cryoablati* OR rfa)) AND (MH "Cardiac-Gated Imaging Techniques" OR MH "Diagnostic Imaging" OR MH "Magnetic Resonance Imaging+" OR MH "Tomography, X-Ray Computed+" OR MH "Tomography, Emission-Computed+" OR TI (imaging OR image* OR (magnet* N2 resonan*) OR ceMRI OR ceMR OR mri OR nmr OR mr OR (comput* N2 tomograph*) OR ct OR ((cat OR pet) N2 scan*) OR cmr OR cmri OR ((positron* OR emissi*) N2 tomogra*)) OR AB (imaging OR image* OR (magnet* N2 resonan*) OR ceMRI OR ceMR OR mri OR nmr OR mr OR (comput* N2 tomograph*) OR ct OR ((cat OR pet) N2 scan*) OR cmr OR cmri OR ((positron* OR emissi*) N2 tomogra*))) NOT (MH animals+ NOT MH humans+)

**Google scholar**

"ventricle|ventricular|rvot|lv|rv|lvot tachycardia|tachyarrhythmia|arrhythmia" ablation|cryoablation|rfa imaging|image|"magnetic resonance"|mri|nmr|"computed|computer tomography"|"positron emission tomography"

**Appendix 4.:** Newcastle- Ottawa Quality Assessment Scale

Note: A study can be awarded a maximum of one star for each numbered item within the Selection and Exposure categories. A maximum of two stars can be given for Comparability.

**Selection**

1) Is definition of NCDs adequate?

a) Yes, according to a clear and widely used definition *****

b) Yes, eg record linkage or based on self-reports

c) No description

2) Representativeness of the cases

a) Consecutive or obviously representative series of cases *****

b) Excluded cases are random *****

c) No description of the excluded cases or potential for selection biases or not stated

3) Comparison with a reference group

a) The results are compared with a reference from community or with the status of the cases prior to the disease *****

b) The results are compared with the results from other patients

c) No description/no comparison available

4) Definition of reference

a) Individuals with no NCD or sample from general population or the same individuals before NCD suffering*****

b) Non community comparator is described

c) No description of source

**Comparability**

1) Comparability of the results on the basis of the design or analysis

a) The results are described in age and sex sub groups (sex is not applicable for female diseases) *****

b) The results are additionally adjusted for/described in different socioeconomic factors or disease related confounders*****

**Exposure (costs, productivity, households)**

1) Ascertainment of exposure

a) Secure record (e.g. surgical records, hospital records, and administrative records, national…) *****

b) Structured interview were blind to case/control status *****

c) Interview not blinded to case/control status

d) Written self-report or medical record only

e) No description

2) Same method of ascertainment for NCDs and comparators

a) Yes *****

b) No

c) No comparator group exist

3) Non-Response rate

a) All participants included or same rate for both groups or respondents and non-respondents have the same characteristics*****

b) Non respondents described

c) Rate different and no designation

d) Response rate not described
