# Supplementary material for: Pre-procedural image-guided versus non-image-guided ventricular tachycardia ablation—a review
Source: Neth Heart J. 2020 Sep 15;28(11):573–83. doi: 10.1007/s12471-020-01485-z (PMC7596120; doi:10.1007/s12471-020-01485-z)

Supplementary file 1: Sensitivity analysis of studies included in meta-analysis

| **Subgroups by Study Characteristics** | | **Number of studies** | **Proportion**  **(95% CI)** | **I^2^ for heterogeneity** | ***P*-value for heterogeneity** |
| --- | --- | --- | --- | --- | --- |
| **Proportion of VT-free subjects** | | | | | |
| **Median percentage of male population** | ≤90% | 17 | 0.56 (0.48-0.65) | 96.7% | **0.3** |
|  | >90% | 16 | 0.62 (0.55-0.69) | 92.5% |  |
| **Median age** | ≤66 | 16 | 0.62 (0.55-0.69) | 95% | **0.2** |
|  | >66 | 17 | 0.57 (0.52-0.62) | 80.4% |  |
| **Median duration of follow-up** | ≤28 months | 19 | 0.60 (0.54-0.66) | 94.1% | 0.6 |
|  | >28 months | 14 | 0.58 (0.51-0.64) | 88.4% |  |
| **Median Ejection Fraction** | ≤32% | 20 | 0.56 (0.51-0.61) | 84.5% | 0.2 |
|  | >32% | 13 | 0.64 (0.57-0.71) | 94.1% |  |
| **Location** | Europe | 15 | 0.55 (0.46-0.63) | 87.2% | 0.4 |
|  | Asia | 5 | 0.65 (0.53-0.78) | 69.3% |  |
|  | North America | 6 | 0.58 (0.45-0.71) | 96.8% |  |
|  | Multinational | 7 | 0.65 (0.54-0.76) | 98.1% |  |
| **Proportion of VT subjects survived during the follow-up period** | | | | | |
| **Median percentage of male population** | ≤90% | 16 | 0.83 (0.78-0.89) | 95.9% | 0.5 |
|  | >90% | 13 | 0.81 (0.76-0.87) | 86.4% |  |
| **Median age** | ≤66 | 12 | 0.81 (0.75-0.88) | 91.6% | 0.8 |
|  | >66 | 17 | 0.82 (0.76-0.89) | 97.1% |  |
| **Median duration of follow-up** | ≤28 months | 15 | 0.83 (0.77-0.88) | 92.4% | 0.8 |
|  | >28 months | 14 | 0.81 (0.73-0.89) | 97.5% |  |
| **Median Ejection Fraction** | ≤32% | 20 | 0.83 (0.78-0.89) | 97.73% | 0.98 |
|  | >32% | 8 | 0.78 (0.68-0.88) | 88.5% |  |
| **Location** | Europe | 15 | 0.83 (0.77-0.88) | 85.5% | 0.5 |
|  | Asia | 3 | 0.72 (0.64-0.81) | 0% |  |
|  | North America | 6 | 0.87 (0.77-0.97) | 98% |  |
|  | Multinational | 5 | 0.79 (0.67-0.92) | 97.6% |  |
| NOTE: meta-regression was not performed in group of image-guided VT ablation due to small number of available studies (*n*=5) | | | | | |

Supplementary file 2: Funnel plot

1. overall survival
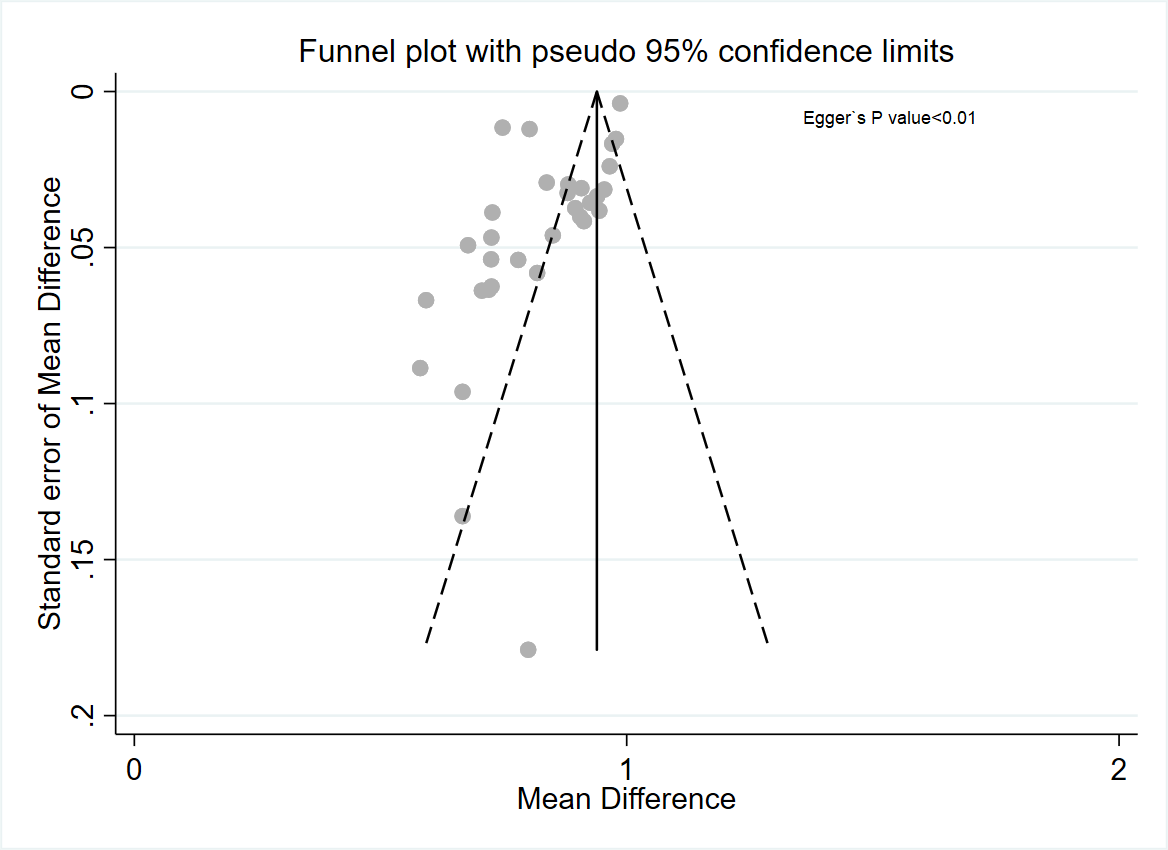

2. VT-free survival


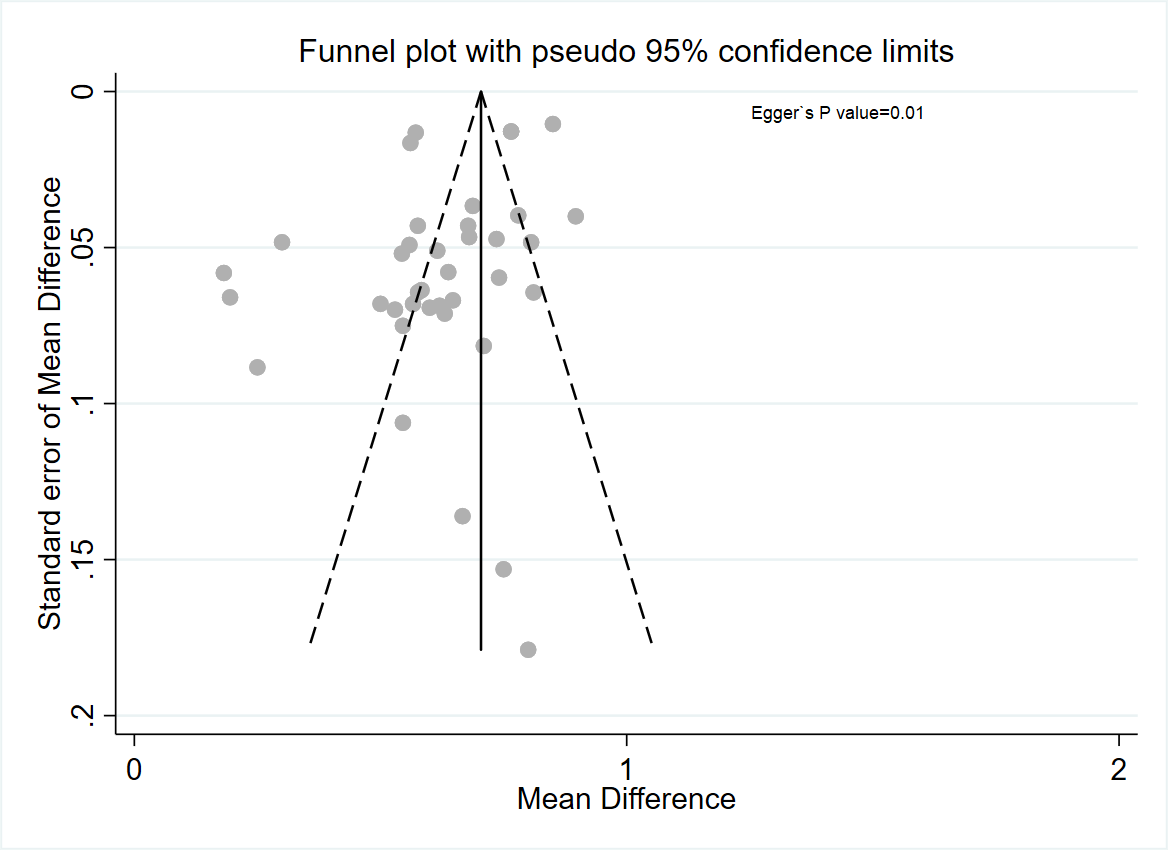

Supplement: Supplementary file 2 — Supplementary files 1 and 2 [file 12471_2020_1485_MOESM2_ESM.docx]
